# 777. Cytomegalovirus: Curriculum-Based Online Education Improves Knowledge, Competence, and Confidence of Physicians

**DOI:** 10.1093/ofid/ofad500.838

**Published:** 2023-11-27

**Authors:** Arun S Nair, Amy Larkin, Iwona Misiuta

**Affiliations:** Medscape Education, TARRYTOWN, New York; Medscape Education, TARRYTOWN, New York; Medscape Education, TARRYTOWN, New York

## Abstract

**Background:**

This study assessed the impact of an online continuing medical education (CME) curriculum regarding CMV infection for obstetricians/gynecologists (OB/GYN) and pediatricians (Peds).

**Methods:**

The educational intervention consisted of 4 online, CME-certified activities in multimedia formats published on a dedicated learning center on Medscape Education from March-July, 2022. Data were collected through January 2023. A repeated pairs pre-/post-assessment study design and a McNemar’s test (P < .05 is considered significant) assessed educational effect. To assess changes in knowledge, competence, and confidence data were aggregated across activities and stratified by learning themes.

**Results:**

The education reached over 65,979 clinician learners, including 20,349 physicians, 9,295 OB/GYN, and 4,407 Peds.

Overall, there were significant improvements in knowledge/competence among OB/GYN and Peds. Specific significant (P < .001) pre-vs-post educational improvements across the curriculum include:

- Knowledge/competence related to the burden of CMV infection (35% OB/GYN, 30% Peds)
- Knowledge/competence related to CMV lifecycle and prevention strategies (25% OB/GYN, 25% Peds)
- Confidence in collaborating with an interprofessional team to discuss CMV risk reduction in pregnant patients (73% OB/GYN, 70% Peds)
- Confidence in understanding the role of CMV serostatus in pregnant patients (78% OB/GYN, 69% Peds)

Remaining educational gaps (% incorrect answers):

- Burden of congenital CMV and CMV infection in pediatric populations (34% of OB/GYN, 35% Peds)
- Universal screening recommendations for congenital CMV in newborn patients (41% OB/GYN, 28% Peds)
- Lifecycle of CMV (34% OB/GYN, 39% Peds)
- Risk reduction strategies for CMV infection in pregnant women (25% OB/GYN, 31% Peds)
- Symptoms and complications pertaining to congenital CMV infection (32% OB/GYN, 35% Peds)

**Conclusion:**

The metrics and outcomes gathered in this assessment are a strong indicator that multimedia curriculum-based online activities on Medscape Education improve the knowledge, competence, and confidence of OB/GYN and Peds related to CMV care strategies. Despite the effectiveness of the education, persistent gaps remain that serve as future educational targets for continuing education.

**Disclosures:**

**All Authors**: No reported disclosures

